# Analysis of the Application Effect of PDCA Cycle Management Combined With Risk Factor Management Nursing for Reducing Infection Rate in Operating Room

**DOI:** 10.3389/fsurg.2022.837014

**Published:** 2022-03-17

**Authors:** Hong Chen, Ping Wang, Qi Ji

**Affiliations:** Operating Room, The First People's Hospital of Lianyungang, Lianyungang, China

**Keywords:** PDCA cycle management, risk factor management, operating room, infection rate, nursing quality

## Abstract

**Purpose:**

To explore the application effect of plan-do-check-action (PDCA) cycle management combined with risk factor management nursing in an operating room.

**Methods:**

A total of 150 surgical patients in our hospital from November 2020 to February 2021 were selected as the conventional group, and 150 surgical patients in our hospital from March 2021 to June 2021 were selected as the research group. The conventional group implemented routine infection management, and the research group implemented PDCA cycle management combined with risk factor management. Detection of pathogenic bacteria, incidence of incision infection, infection control, occurrence of irregular events, and nursing quality in the operating room were observed in the two groups.

**Results:**

The detection rate of Gram-negative bacillus and Gram-positive cocci, infection rate of incision, and total incidence of irregular events in the research group were lower than those in the conventional group (*P* < 0.05). The qualified rate of disinfection of object surface, hands of medical staff and air, and nursing quality scores in the research group were higher than those in the conventional group (*P* < 0.05).

**Conclusion:**

Plan-do-check-action (PDCA) cycle management combined with risk factor management nursing can reduce the detection rate of pathogenic bacteria and infection rate of incision in the operating room, reduce the incidence of irregular events, improve the qualified rate of disinfection, and greatly improve the quality of nursing, which can be considered to be widely used in clinical practice.

## Introduction

An operating room is the main place for disease treatment and emergency rescue of surgical patients. The patient's condition in an operating room is complex and changeable, and has a high risk. An operating room is the place where hospital infection is most likely to occur. In an operating room, air, particles, hands of medical staff, and other factors may become the way of infection for patients (1, 2). Once patients are infected in an operating room, it may cause other diseases, which will affect the therapeutic effect and clinical outcome of an operation, and even threaten the patient's life. At the same time, infection of patients in an operating room will increase hospital dispute rate and increase the workload of medical staff (3, 4). An operating room has a wide range of work, many complicated events, many medical staff, and high risk of infection. Therefore, compared with outpatient and inpatient areas, an operating room has higher requirements for nursing quality management (5). However, at present, there is a big gap between actual infection management status and expected nursing goals in most operating rooms. The quality of infection management in an operating room not only directly affects the life safety of surgical patients but is also closely related to the service quality of the whole surgical system. How to build a high-quality infection control management system, ensure the safety of surgical patients, and improve the quality of operating room environments is the clear goal of operating room managers.

Plan-do-check-action (PDCA) cycle management is an effective management tool. The PDCA cycle divides quality management into four stages: plan, do, check, and action; it also divides quality management into eight steps: asking questions, setting goals, drawing up plans, implementing plans, checking results, finding out problems, solving problems, and putting forward new plans (6). In PDCA cycle management, every link is closely linked, and it is implemented repeatedly without stopping, so management objectives can be achieved step by step (7). PDCA cycle management is a scientific and comprehensive working procedure of quality management. Applying PDCA cycle management to nursing management can obviously improve nursing quality and reduce shortage in management scheme, so as to obtain better overall hospital quality (8). Risk factor management refers to the process of minimizing the probability of risk occurrence by studying the occurrence law of risk and thinking about how to control risk and deal with risk factors in the management system (9). Applying risk factor management to nursing work by risk identification and risk assessment can improve the risk awareness of medical staff, effectively prevent the occurrence of risk factors, reduce harm to patients and hospitals, reduce economic losses, and obtain greatest security (10).

At present, PDCA cycle management and risk factor management have been widely recognized by people in the field of nursing management at home and abroad. However, the effect of applying the above two management methods to infection care in operating rooms is worth considering by medical staff. PDCA cycle management combined with risk factor management nursing is implemented in infection management of operating rooms in our hospital. Detailed intervention methods and intervention effects are summarized as follows.

## Materials and Methods

### Research Object

A total 150 surgical patients in our hospital from November 2020 to February 2021 were selected as the conventional group, and 150 surgical patients in our hospital from March 2021 to June 2021 were selected as the research group. Inclusion criteria were: ① no serious organ diseases, ② no severe anemia, and ③ normal intelligence. Exclusion criteria were: ① an infection existed before admission, ② complicated with immune system diseases, ③ complicated with malignant tumor, and ④ poor compliance. The general data of the two groups were balanced and comparable (*P* > 0.05). See Table 1 for specific general data.

**Table 1:** Comparison of general data between the two groups (*n*, %).

**Group**	**Gender**		**Age (years)**				
	**Male**	**Female**	**<40**	**≥40**	**Class I incision**	**Class II incision**	**Class III incision**
Conventional group (*n* = 150)	89 (59.33%)	61 (40.67%)	68 (45.33%)	82 (54.67%)			
Research group (*n* = 150)	93 (62.00%)	57 (38.00%)	61 (40.67%)	89 (59.33%)			
*χ^2^*value	0.224	0.666	
*P*-value	0.636	0.414	

### Research Methods

(1) The conventional group implemented routine infection management, including environmental disinfection of operating room, sterilization of instruments, prevention of incision infection, disinfection and isolation, hand hygiene of medical staff, and management of sterile goods, etc.
(2) The research group implemented PDCA cycle management combined with risk factor management. The specific nursing methods were as follows.

Risk management nursing: we investigated the risk factors of infection in the operating room, such as nurses' lack of awareness of infection risk, inadequate management, personnel turnover, and other factors; we formulated a reasonable and preventable management system according to identified infection risk factors, summarized the risk factors, and gave early warning and feedback to doctors in time for factors that were highly suspected to cause surgical infection. A hospital sense control team was established, which was composed of nurses with working experience > 10 years, and nursing staff with the title of chief nurse or above. The team members discussed the influencing reasons of risk factors together and adjusted the management plan appropriately. We updated various infection rules and regulations according to the actual situation of the hospital, including disinfection, hand washing, sanitation, visit, waste treatment, and other projects; we formulated an infection monitoring record list; regular inspections and supervision of disinfection and isolation of the operating room were carried out, and infection management was carried out under the guidance of PDCA cycle management.

Plan-do-check-action (PDCA) cycle management: ① plan: observed the management status of the operating room, find out core problems at present, establish an infection management plan for the operating room with reference to “Hospital Infection Management Standard”, and formulate an investigation table of nursing quality in the operating room, including environmental management, nursing safety, item management, disinfection and isolation, etc; ② do: the department regularly organized training to urge medical staff to master the knowledge of hand hygiene, disinfection and isolation, dressing in the operating room and medical wastes, and implement various rules and regulations; detailed instructions of seven washing techniques and precautions for hand disinfection were pasted above the sink, and monitor whether the operating staff strictly followed the operating rules of hand washing and disinfection; The operating room was scientifically arranged, the passage of medical staff was reasonably arranged, the number of visitors to an operation was limited, the sterile operating room, the general operating room, and the infection operating room were strictly distinguished, inter-surgery was prohibited, the flow of items was controlled, and the items and equipment in the operating room were fixed and did not frequently change locations; a chlorinated disinfectant was used to wipe the surface of objects in and the floor of the operating room to ensure that surgical items were dry and sterile. The area above the shoulder of the operator and below the belly button was the area with bacteria. The sterile table cloth sag > 30 cm should be replaced immediately after being touched; the sterilized surgical items were managed by a dedicated person, the sterile validity period of disposable articles should be checked, the sealing property should be checked, and medical wastes should be classified and disposed according to the medical waste specification after use; ③ check: key links of infection control, such as surgical instruments, sterile materials, disinfectants were regularly checked, and various infection management tasks were filled in the supervision record form; the operating room was comprehensively reviewed according to the operating room nursing quality inspection form. In the check, the hospital infection office personnel participated in the supervision, analyzed existing infection prevention and control problems, and negotiated to solve the problems. Methods such as theoretical assessment, operational skill assessment, and random questioning were used to assess the knowledge of nurses on the prevention of infection in the operating room, and spot-checked on infection control has been implemented; ④ action: summarized the problems and risk factors of infection in operating room, found out the inadequacies of management from an objective point of view, adjusted the management plan according to the characteristics of departments, formulated targeted solutions, and rectified deficiencies one by one. Unsolvable problems and improvement goals were put into the next PDCA cycle mode, so that the infection control work in the operating room was continuously and systematically monitored.

### Observation Index

① Blood 2 ml was taken from patients in both groups 24 h after operation, and pathogen culture was conducted to observe the detection rate of pathogenic bacteria in the two groups of patients.
② Criteria for determining infection referred to the hospital infection criteria (11), and infection rates of various surgical incisions in the operating room were observed.
③ After the operation, sterile cotton swab was dipped in a sampling solution and evenly smeared on the surface of the object 5 cm × 5 cm back and forth. The cotton swab was placed in a test tube with 5 ml sampling solution and vibrated 80 times. After proper dilution, it was inoculated on a common nutrient agar plate to count the viable bacteria. Six objects were sampled on the surface at a time, with a sampling area of 100 cm^2^. The sampling method of medical staff's hands was the same as that of the objects, with six people being sampled at a time, and the sampling area was 60 cm^2^ of both hands. Natural sedimentation method was used for environmental sampling, and the diameter of the plate was 9 cm. Five sampling sites were arranged in each operating room, which were exposed for 30 min, with a swab dipped in the sample solution spread evenly over a plate. The qualification of the operating room disinfection was determined according to the sanitary standards for hospital disinfection (12): the operating environment belongs to Class I environment, with the total number of bacterial colonies in the air ≤ 10 cfu/m^3^, the total number of bacterial colonies on the surface of objects ≤ 5 cfu/m^2^, and the total number of bacterial colonies on the hands of medical staff ≤ 5 cfu/m^2^. Disinfectant for sterilization in use: aseptic growth; the amount of bacteria contaminated by skin and mucosal disinfectant in use: ≤ 10 cfu/ml; the amount of bacteria contaminated by other disinfectant in use: ≤ 100 cfu/ml; no pathogenic bacteria detected was considered qualified, and no bacterial growth was considered qualified.
④ Irregular events such as sterilization of instruments were not standardized, hand hygiene was incomplete, and frequent personnel activities and improper disposal of surgical wastes were recorded in the process of surgical nursing.
⑤ Members of the nursing quality monitoring team used the operating room nursing quality evaluation table made by the hospital, including environmental management, nursing safety, item management, disinfection,and isolation. There were 10 items in each dimension, which were recorded as 0~10 points, and total score was 100 points; the higher the score, the better the nursing quality.

### Statistical Methods

The SPSS 22.0 software was used for analysis. The measurement data was mean ± standard deviation (*x* ± s), the comparison was made by *t*-test, the count data was ratio (%), and the comparison was made by χ^2^ test.

## Results

### Detection of Pathogenic Bacteria in the Two Groups

Detection rates of Gram-negative bacillus and Gram-positive cocci in the research group are lower than those in the conventional group (*P* < 0.05), as shown in Table 2.

**Table 2:** Detection of pathogenic bacteria in the two groups (*n*, %).

**Group**	**Gram-negative bacillus**	**Gram-positive cocci**
Conventional group (*n* = 150)	14 (9.33%)	11 (7.33%)
Research group (*n* = 150)	5 (3.33%)	3 (2.00%)
*χ^2^*value	4.551	4.795
*P*-value	0.033	0.029

### Incidence of Surgical Incision Infection in the Two Groups

The rate of incision infection in the research group is lower than that in the conventional group (*P* < 0.05), as shown in Table 3.

**Table 3:** Incidence of surgical incision infection in the two groups (*n*, %).

**Group**	**Class I incision**	**Class II incision**	**Class III incision**	**Total incision infection rate**
Conventional group (*n* = 150)	3 (2.00%)	8 (5.33%)	6 (4.00%)	17 (11.33%)
Research group (*n* = 150)	1 (0.67%)	3 (2.00%)	2 (1.33%)	6 (4.00%)
*χ^2^*value				5.698
*P*-value				0.017

### Infection Control in the Two Groups

Qualified rates of disinfection of object surface, hands of medical staff, and air in the research group are higher than those in the conventional group (*P* < 0.05), as shown in Table 4.

**Table 4:** Infection control in the two groups (*n*, %).

**Group**	**Qualified rate of disinfection**				
	**Object surface**	**Hand of medical staff**	**Air**	**Sterile articles**	**Disinfector**
Conventional group (*n* = 150)	138 (92.00%)	128 (85.33%)	135 (90.00%)	149 (99.33%)	146(97.33%)
Research group (*n* = 150)	146 (97.33%)	141 (94.00%)	144 (96.00%)	150 (100.00%)	149(99.33%)
*χ^2^*value	4.225	6.080	4.147	1.003	1.831
*P*-value	0.040	0.014	0.042	0.317	0.176

### Occurrence of Irregular Events in the Two Groups

The total incidence of irregular events in the research group is lower than that in the conventional group (*P* < 0.05), as shown in Table 5.

**Table 5:** Occurrence of irregular events in the two groups (*n*, %).

**Group**	**Sterilization of instruments was not standardized**	**Hand hygiene was incomplete**	**Frequent personnel activities**	**Improper disposal of surgical wastes**	**Total incidence rate**
Conventional group (*n =* 150)	3 (2.00%)	4 (2.67%)	6 (4.00%)	2 (1.33%)	15 (10.00%)
Research group (*n* = 150)	1 (0.67%)	1 (0.67%)	2 (1.33%)	1 (0.67%)	5 (3.33%)
*χ^2^*value					5.357
*P*-value					0.021

### Nursing Quality in the Operating Room for the Two Groups

**Figure 1:**
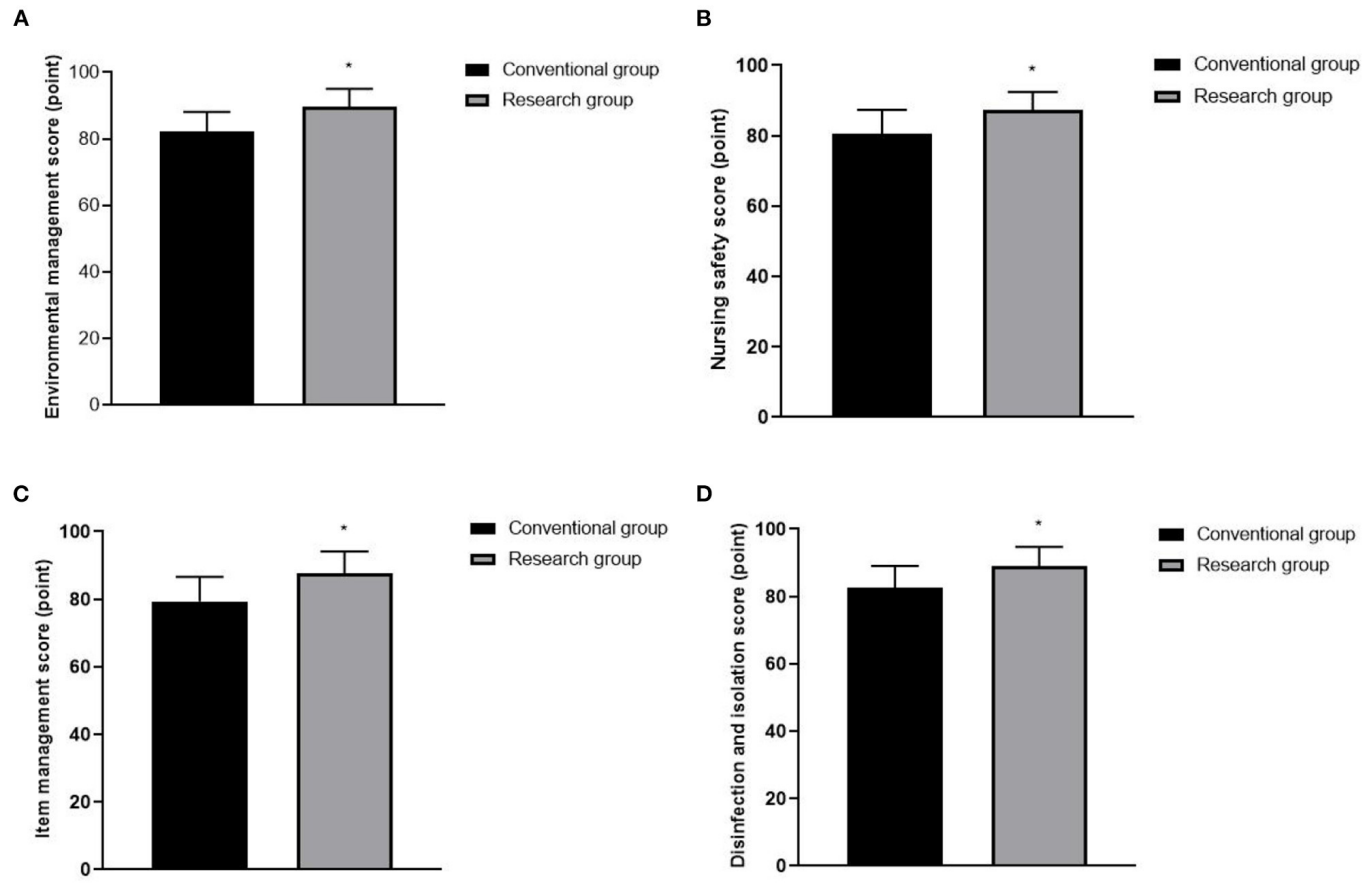
Nursing quality in operating room for the two groups. **(A)** Environmental management score. **(B)** Nursing safety score. **(C)** Item management score. **(D)** Disinfection and isolation score. Compared with the conventional group, *P < 0.05.

Nursing quality scores of the research group are higher than those of the conventional group (*P* < 0.05), as shown in Figure 1.

## Discussion

Studies have shown that additional mortality rate caused by patients infected in hospitals is 4~33%, which not only seriously endangers the life and health of patients but also increases hospitalization days, cost of treatment, and doctor-patient conflicts, causing certain negative impacts on patients and the society (13). With the improvement of people's health awareness, people have a deeper understanding of medical information, patients' requirements for medical care are gradually increasing, and medical institutions are paying more and more attention to bacteria. An operating room is one of the main departments of hospital infection, and there are many risk factors leading to patient infection, such as unqualified air disinfection in the operating room, incomplete sterilization of surgical instruments, nonstandard placement of surgical items, weak awareness of infection prevention and control among medical staff, and frequent walking of visitors (14, 15). The management of infections in operating rooms cannot be ignored for the surgical effect of patients, and reducing infections has become the key concern of operating room services.

Risk management is a management concept with risk prevention awareness. By summarizing the accumulated experience of medical staff for many years, we can identify risk factors that may lead to unsafe nursing events, formulate targeted intervention measures according to screened high-risk factors, and regularly evaluate changes in risk factors, explore items that need improvement, and adjust intervention schemes, so as to avoid the occurrence of adverse phenomena (16, 17). In this management mode, risk factors are basically factors that can be intervened with and changed, and can be used as the focal point of risk control. By forward-looking analysis, nurses can implement corresponding treatment measures, thus reducing the probability of unsafe events, improving patients' satisfaction, reducing medical disputes, and meeting patients' medical needs with best nursing service (18, 19). In addition, PDCA cycle management is a step-by-step process of dynamic cycle according to the specific situation of each department, making targeted goals and plans, implementing PDCA small cycle and large cycle, gradually improving the quality of nursing work under repeated plan-do-check-action, and, finally, achieving the ideal goal (20). PDCA cycle is a comprehensive cycle, the four stages are inseparable, and the cycle is continuous, with standardization and institutionalization (21). In the PDCA cycle, the small cycle is the decomposition and guarantee of the implementation of the large cycle. Every time the PDCA small cycle is implemented, some management results will be achieved, management level will be improved, and work quality will be improved. After the small cycle ends, it is necessary to summarize, analyze, and update the plan and adjust the scheme, and then enter the next small cycle, which urges the large cycle to keep moving forward (22–24).

According to the actual situation of the operating room, we carried out PDCA cycle management combined with risk factor management nursing. The main intervention items included risk factor assessment, operating room environmental management, goods management, hand hygiene management, aseptic management, and other measures. After the intervention, the detection rate of pathogenic bacteria, rate of incision infection, and total rate of occurrence of irregular events were lower than those in patients with routine nursing, and disinfection qualified rate and nursing quality score were higher. Risk factor management can supervise and restrict every PDCA link in infection control in operating rooms, and form a reciprocating cycle of risk assessment and risk control, factors that easily lead to infection can be evaluated more objectively, and corresponding interventions targeted at potential risks can be implemented while ensuring the disinfection quality of operating rooms, which has a positive effect on reducing infection in operating rooms and improving the quality of operation (25). The combination of the two management measures could create a virtuous cycle of management improvements in the operating room targeting infection risk factors, and the infection management time of nursing staff can be shortened. At the same time, in the development of PDCA cycle combined with risk factor management, we strengthen the supervision and management of infection control through various forms of disinfection qualification rate assessment and bacterial examination, enhance the disinfection consciousness and aseptic operation consciousness of operating room staff, and realize the management concept of prevention first, thus significantly improving the effect of disinfection and sterilization, reducing irregular events in the operating room, and building a safe operating environment, which is conducive to wound healing of surgical patients and achieving good results in infection nursing in the operating room.

## Conclusion

To sum up, PDCA cycle management combined with risk factor management nursing can reduce the detection rate of pathogenic bacteria and rate of incision infection in operating rooms, reduce the incidence of irregular events, improve the qualified rate of disinfection, and greatly improve the quality of nursing, which can be considered to be widely used in clinical practice.

## Data Availability Statement

The original contributions presented in the study are included in the article/supplementary material, further inquiries can be directed to the corresponding author.

## Ethics Statement

The studies involving human participants were reviewed and approved by the First People's Hospital of Lianyungang. The patients/participants provided their written informed consent to participate in this study.

## Author Contributions

HC is mainly responsible for the writing of articles. PW is mainly responsible for the design of the research and the statistics of the results. QJ is mainly responsible for the guidance of the entire research process. All authors contributed to the article and approved the submitted version.

## Conflict of Interest

The authors declare that the research was conducted in the absence of any commercial or financial relationships that could be construed as a potential conflict of interest.

## Publisher's Note

All claims expressed in this article are solely those of the authors and do not necessarily represent those of their affiliated organizations, or those of the publisher, the editors and the reviewers. Any product that may be evaluated in this article, or claim that may be made by its manufacturer, is not guaranteed or endorsed by the publisher.
